# Is there a relationship between moral competencies and the formation of professional identity among nursing students?

**DOI:** 10.1186/s12912-020-00440-y

**Published:** 2020-06-10

**Authors:** Sahar Haghighat, Fariba Borhani, Hadi Ranjbar

**Affiliations:** 1 School of Nursing & Midwifery, Shahid Beheshti University of Medical Sciences, Tehran, Iran; 2 Nursing Research Center, Golestan University of Medical Sciences, Gorgan, Iran; 3 Medical Ethics and Law Research Center of Shahid Beheshti University of Medical Sciences, Tehran, Iran; 4 Mental Health Research Center, Psychosocial Health Research Institute, Iran University of Medical Science, Shahid Mansouri st, Niyayesh St, Sattarkhan Ave, Tehran, 1445613111 Iran

**Keywords:** Moral development, Professional identity, Students, Nursing, Education, Competency

## Abstract

**Background:**

Moral competencies are essential for nursing work. Professional identity is a set of values and beliefs that a person has about her/his job, which includes moral values as well. The development of moral competencies and formation of professional identity in nursing students occurs mainly during their college years. The aim of this study was to investigate the relationship between moral competencies and the formation of professional identity among nursing students.

**Methods:**

This study was designed as a descriptive-correlational study. The study population was consisted of nursing students who were enrolled in nursing schools at the time of the study. Two hundred and twenty-one nursing students completed the study tools. The research tools were a demographic questionnaire, Moral Development Scale for Professionals (MDSP), and Professional Identity Scale for Nursing Students (PISNS).

**Results:**

The mean (SD) of MDSP and PISNS scores was 45.69 ± 5.90 and 55.61 ± 12.75, respectively. There was a significant statistical relationship between MSDP and PISNS scores (*p* < 0.05). A significant equation was found (f (2, 218) = 16.68, *p* < 0.001) with an R2 of 0.113. The MSDP scores increased 0.136 for each score of PISNS, and married students had 2.452 scores higher than single students.

**Conclusions:**

The positive correlation between the formation of professional identity and development of morality in nursing students indicates that by strengthening students’ professional values, their moral competencies may develop positively.

## Background

Moral competence is the capacity to make decisions and judgments, which are moral based on internal principles and to act according to them [1]. It is also the ability of using moral principles in action and workplace to cope with problems and use in conflict resolution based on inner moral principles rather than external social expectations [2, 3]. It is a needed competence for nurses to deliver moral care provision [4]. Professional identity is defined as “the attitudes, values, knowledge, beliefs and skills shared with others within a professional group” [5]. The formation of professional identity is critical to the effective and safe practice of all health professions, including nurses. The development of professional identity is a continuous process and it is critical to the effective and safe practice of all health professionals, including nurses. The development of professional identity is a continuous process. The process is influenced by factors such as professional socialization and experiences in practice [6]. A significant amount of the development of professional identity occurs during college years [7, 8].

Providing high quality and safe care needs ethical nursing practice, which is increasingly challenging by technological and technical growth in health systems [9]. Therefore, new strategies are needed to support the nurses’ moral care provision [10]. One of the recommended strategies is strengthening professional competencies. The assumption of this strategy is that people who are more professionally competent can do their duties more professionally.

Acquisition of moral competencies happens in a process called moral development. Moral development is defined as the change in moral behavior over time [11]. Most of moral development process is happening during college years. However, recent studies have shown that the degree of moral competence development in all students is not equal and satisfactory [12, 13]. Moral behavior is the capacity to distinguish between right and wrong and to act accordingly [14]. Nurses need to reach to high levels of moral competence because most of the procedures and decisions in their daily practice have moral dimensions.

While the nursing shortage became a global problem, many nursing students, who should replace with retired nurses, do not have the required moral competencies to deal with workplace issues at the time of graduation [15]. It is the responsibility of nursing education institutes to develop professional competencies, especially moral competencies in their students.

Nursing education faces several challenges such as the high rate of retirement of registered nurses and nursing educators [16, 17], low level of competencies among nursing graduates [18] including moral competence [11, 19, 20]. Considering the commission and the Global Health Workforce Alliance report which indicates that professional education has not kept up the speed of health care challenges [21], these challenges will be worsened in the future decade. It is also because the demands for nurses will increase globally with the aging societies, and new ethical dilemmas will rise by technological innovations [22]. Due to these challenges, the training of nurses who can provide moral care is increasingly challenging.

Moral competence is an abstract concept, and it consists of cognitive, affective and behavioral components [23]. While nursing programs have ethical courses, evaluation of their outcomes is not easy. At the beginning of their career, measuring nurses’ moral competences is not easy, as well. How can nurses’ professional competencies be more effectively developed? And how can their professional competences be predicted at the start of their career? The results of a recent study showed that there is probably a relationship between moral development and the formation of professional identity in nursing students [13]. Their findings indicate that the development of morality may be connected to the formation of professional identity. Professional identity is defined as a self-perception about the profession based on attitudes, beliefs, feelings, values, motivations, and experiences [24, 25]. The formation of professional identity, is an essential factor in increasing self-confidence, feeling of belonging to the profession and establishing interpersonal communication among nurses [26, 27].

In the literature review, we did not find any study that assessed the correlation between the two constructs of moral development and professional identity in nurses or nursing students. Ranjbar, Joolaee [13] discussed that maybe there is a correlation between professional identity formation and development of morality in nursing students. They concluded that in order to have higher moral practice in nursing students, nursing instructors should promote the students’ professional identity. Bliss, Baltzly [28] argued that the nurse’s professional identity associates their practice with the relationship between them and patients, which morality is one of its most important dimensions [29]. Some scholars argued that professional values, including moral values, are essential in the formation of professional identity in nursing students [30, 31]. Also, Dehghani, Mosalanejad [32] found that individual character and responsibility are two important factors affecting professional ethics in nursing practice.

While one of the most important aims of all education systems is the development of morality in their graduates [33], some of them failed to reach to this aim [34]. Failure to develop the morality in nursing students can have bad consequences. There are many factors that influence the moral development of nursing students. Professional identity is a potential one which was mentioned in the literature. Since the measurement of professional identity is more accessible than moral development, it can be used in prediction of the moral development of nurses. Furthermore, if there is a relationship between these two variables, then nursing programs can benefit from helping the formation of professional identity in the development of moral competencies. Based on this hypothesis, the purpose of this study was to investigate the relationship between the development of moral competencies and the formation of professional identity in nursing students.

## Methods

### Study design

This was a descriptive-correlational study.

### Sample and setting

The study was carried out at three nursing schools in Iran’s capital, Tehran. The sample was recruited by random stratified sampling from undergraduate nursing students. The minimum sample size was calculated using G*power software. The criteria for determining sample size were alpha = 0.05, power = 0.80, and an effect size of 0.25 with 20 % of drop-out rate (*n* = 240). The number of samples in each school was calculated based on the number of students studying at the time of sampling.

### Measurements

The research tools were a demographic questionnaire, Moral Development Scale for Professionals (MDSP) [35], and Professional Identity Scale for Nursing Students (PISNS) [36].

### The moral development scale for professionals (MDSP)

The Moral Development Scale for Professionals (MDSP) has twelve items and consists of four dimensions, including Authoritative standards, Public meaning, Moral practice, and Common values. Each item scored on 5-point Likert from 1 (Strongly Disagree) to 5 (Strongly Agree). The MDSP scores range from 12 to 60, with higher scores indicating higher moral development. Skisland, Bjornestad [35] reported construct validity and Cronbach’s alpha coefficient (0.67) for it. The MDSP was translated to Persian by a bilingual individual whose first language was Persian. It was then back-translated to English by a bilingual person whose mother language was English. The face and content validity of the questionnaire was assessed by 10 nursing experts, who examined the clarity of the translated items. Cronbach’s alpha was calculated to assess internal reliability (α = 0.74).

### The professional identity scale for nursing students (PISNS)

The Professional Identity Scale for Nursing Students (PISNS) has 17 items [36]. It consists of five dimensions, including 1) social modeling, 2) independence of career choice, 3) social comparison and self-reflection, 4) benefit of retention and the risk of turnover and 5) Professional self-image. Scale items are scored on a 5-point Likert from Strongly Disagree [1] to Strongly Agree [5]. Construct validity showed five factors model explaining 58.9% of the total variance. Cronbach's alpha and split-half reliability was 0.83 and 0.84 respectively. The PISNS scores range from 17 to 85, with higher scores indicating higher professional identity. The face and content validity of the questionnaire were assessed by 10 nursing experts. They examined the clarity and simplicity of the translated items. Cronbach’s alpha was calculated to assess internal reliability (α = 0.89).

### Data gathering

The researchers took written permission from the schools’ management and presented it to sample sites. In each School, all students were asked to participate in the study. Two hundred and forty-two questionnaires were distributed among students who were willing to participate in the study. The research objectives and the confidentiality of information were explained for all study subjects, and all of them verbally declared their consent.

### Statistical analysis

Data were analyzed using SPSS 16. The Kolmogorov–Smirnov test shows that the distribution of MDSP and PISNS scores was normal. Pearson correlation test and linear regression were used to describe the relationship between the formation of professional identity and moral development in nursing students.

## Results

Two hundred and twenty-one (90%) of the 242 distributed questionnaires were returned from study subjects. The demographic characteristics of the study subjects are presented in Table 1.

**Table 1:** Demographic characteristics of study subjects (*N* = 221)

Variable		Number	Percentage
Sex	Male	109	49.3
	Female	112	50.7
Marital status	Single	186	84.2
	Married	35	18.8
Part-time nursing work	No	80	36.2
	Yes	141	63.8

The mean (SD) of MDSP and PISNS scores was 45.69 ± 5.90 and 55.61 ± 12.75, respectively. There was a significant statistical relationship between MSDP and PISNS scores (*p* < 0.001). There was no statistically significant relationship between the “social modeling” dimension of professional identity scale and “ethical practice” dimension of the moral development questionnaire. Other dimensions of the two scales were correlated (Table 2).

**Table 2:** The correlation between professional identity and moral development dimensions

Correlation	Authoritative standards	Public meaning	Moral practice	Common values	Moral Development
Social modeling	*r* = 0.322 *p* < 0.001	*r* = 0.208 *p* = 0.002	*r* = 0.138 *p* = 0.04	*r* = 0.216 *p* = 0.04	*r* = 0.290 *p* = 0.04
Independence of career choice	*r* = 0.208 *p* = 0.002	*r* = 0.198 *p* = 0.003	*r* = 0.152 *p* = 0.02	*r* = 0.283 *p* < 0.001	*r* = 0.261 *p* < 0.001
Social comparison and self-reflection	*r* = 0.297 *p* < 0.001	*r* = 0.270 *p* < 0.001	*r* = 0.260 *p* = 0.002	*r* = 0.349 *p* < 0.001	*r* = 0.354 *p* < 0.001
Benefit of retention and the risk of turnover	*r* = 0.327 *p* < 0.001	*r* = 0.230 *p* = 0.001	*r* = 0.127 *p* = 0.06	*r* = 0.183 *p* = 0.006	*r* = 0.288 *p* < 0.001
Professional self-image	*r* = 0.279 *p* < 0.001	*r* = 0.195 *p* = 0.004	*r* = 0.087 *p* = 0.19	*r* = 0.153 *p* = 0.02	*r* = 0.237 *p* < 0.001
Professional identity	*r* = 0.303 *p* < 0.001	*r* = 0.257 *p* < 0.001	*r* = 0.183 *p* = 0.006	*r* = 0.303 *p* < 0.001	*r* = 0.334 *p* < 0.001

Multiple linear regression was calculated to predict MSDP Scores based on PISNS scores, age, sex, part-time nursing work, and marital status. The relationships between age, sex, and part-time nursing work were not significant (*P* > 0.05). A significant equation was found (f (2, 218) = 16.68, *p* < 0.001) with an R^2^ of 0.113. The MSDP scores increased 0.136 for each score of PISNS, and married students had 2.452 scores higher than single students (Table 3). Based on the results of Table 3 there was a positive correlation between the formation of professional identity and moral development in nursing students. Based on this result the moral competence of nursing students increased by the rise of professional identity.

**Table 3:** Multiple linear regression analysis for MSDP scores (stepwise method)

Model	Unstandardized Coefficients		Standardized Coefficients	t	Sig.
	B	Std. Error			
Constant	35.314	1.683		22.05	0.00
PISNS Score	0.136	0.030	0.293	0.474	0.00
Marital Status (being married)	2.452	1.057	0.157	0.320	0.021

## Discussion

The aim of this study was to determine the correlation between the formation of professional identity and the level of moral development in nursing students. Results showed that there was a significant correlation between moral development scale and professional identity formation scale scores. Based on our result, the formation of professional identity influences students’ moral development. Therefore, by helping the formation of professional identity in nursing students, it can be expected that moral competencies will be more developed in them.

Moral development and professional identity separately were the subjects of several studies, but the correlation between them was not assessed before. In a study conducted by Rahimaghaee, Nayeri [37] a significant correlation between inner commitment to work morally and nurses’ professional identity was found. The result of previous studies showed that having a positive professional identity was related to higher job satisfaction and job retention [38–40]. This relationship indicates that the growth of professional identity may be related to increasing the interest to the profession or vice versa. While the literature related to the correlation between job satisfaction and moral competencies is limited, the results from a research showed that there could be a relationship between these two variables [28]. We did not find any studies that assess the relationship between moral development and professional identity formation.

Health care providers need high level of moral competencies to face growing moral issues in their work environments. Nurses are the front lines of healthcare delivery [41] and they need to have enough moral competencies to deliver high-quality care [42]. To understand moral competence, we must first understand the components of it. The componentss of moral competence are (a) A system of norms; (b) a moral vocabulary; (c) moral cognition and effect; (d) moral decision making and action; and (e) moral communication [43]. The system of norms derives from personal, social and professional values [23], and nursing education has a significant effect on the development of professional values in nursing students [44].

The professional value system is one important part of professional identity. In the process of formation of professional identity, the system of norms and values forms in nursing students and nurses. Nursing Students learn these values from instructors and other nurses as well as other students. Their experiences from college and hospitals has also a significant role in the development of professional values [45]. Development of moral competencies needs awareness and obligation to nursing professional values. Professional values help nurses to (a) identify moral problems, (b) reason and judge based on values and (c) act according to them [23]. Professional values have a crucial role in the moral function of nurses. However, the focus of nursing education programs is on increasing students’ knowledge and skills. Weis and Schank [46] argued that professional values are the key to professional development. They suggested a model for professional development. They also offered 11 presuppositions concerning value formation. Based on their model value formation is a process which has critical stages. The students should be ready and targeted experiences should be provided for them. Personal values also affect the process. They also discussed that the full embodiment of professional values might be a distinguishing factor between professional and technical nurses. This result is in line with the results of [12, 13] which categorized the level of moral development in nursing students and related it with their level of formation of professional identity.

Ranjbar, Joolaee [13] argued that the development of morality in nursing students is related to the formation of professional identity. Nursing students and nurses develop and internalize professional values in a process which can be influenced by various factors. Nursing schools and education system have an irreplaceable effect on this process. Nursing schools are the first place where professional values ​​are introduced to students. If schools focus on increasing knowledge and skills merely, graduated nurses may not have proper internalized professional values, which include moral and ethical values. The emphasis on professional values ​​since the beginning of studies helps students to develop needed competencies for work in accordance with moral and professional values.

### Study limitations

While investigating the correlation between these two constructs for the first time is one of the strengths of the present study, the limitation associated with correlational studies is that they don’t prove causation. We recommend interventional studies to assess this correlation more carefully. Other limitation of this study was that the study subjects were recruited from just one city. Including other cities and more universities would help generalizability of the results. Another limitation is related to instruments. Using self-reported instruments to measure moral development and professional identity may also have affected the results. Future studies are needed to evaluate these two variables more objectively.

### Implications for nursing education and practice

Nursing managers can use our results in practice. Having High moral competencies is a good standard that every nurse needs to have. By measuring the extent of professional identity formation and moral development of nurses, nursing managers can employ more qualified nurses. Also, they can use strategies to improve nurse’s professional identity in order to increase the moral practice. New researches can be done in order to identify more effective factors in the process of professional identity formation and moral development, which are two important issue in nurses. We also recommend interventional studies to confirm the causal relationship between these two constructs. Since moral development in nursing students is one of the most important goals of the education system, nursing educators and curriculum developers can benefit from our results.

## Conclusions

The study results showed that the moral competencies of nursing students is related to their professional identity formation. Nursing shortage may cause hiring nurses without enough selection criteria. Nurses must be at the highest level of moral development in order to be able to do their job ethically. Nursing schools can provide a curriculum based on professional values to improve the status of professional identity. Based on our results, nurses with well-formed professional identity had higher levels of moral development. This result indicates that in order to develop morality in nursing students, educators can use techniques, which effect on the formation of professional identity. Furthermore, in the the practice, managers can grow their employee’s skills by emphasizing on the professional identity of nurses. Another implication of these results is in research which it can use to investigate this relationship more carefully. Based on the results of our current research and previous studies, we believe that identifying self as a nurse will affect the moral performance of nurses.

## Data Availability

All data will be available on request. Everyone can request the data. To gain access, data requestors will need to sign a data access agreement. The data is available for any purpose. All applications should be sent to Ranjbar.h@iums.ac.ir. All requests will be answered within a maximum of 1 month by email.

## Abbreviations

MDSP: Moral Development Scale for Professionals
PISNS: Professional Identity Scale for Nursing Students
